# Mechanism of inhibition of TLR4/NFκB/NLRP3 inflammatory pathway against AD based on the network pharmacology of Erjing Pills

**DOI:** 10.1097/MD.0000000000039392

**Published:** 2024-08-23

**Authors:** Chen Zhang, Mingjing Lu, CunNeng Li, Chao Qi, Qian Lin, LiPing Huang, Hailing Ding

**Affiliations:** a School of Medical, Qilu Institute of Technology, Jinan, China; b School of Pharmacy, Jiangxi University of Chinese Medicine, Nanchang, China; c Key Laboratory of TCM Pharmacology of Jiangxi Province, Nanchang, China.

**Keywords:** Alzheimer disease, Erjing Pills, molecular docking, network pharmacology, TLR4/NFκB/NLRP3 inflammatory pathway

## Abstract

Alzheimer disease is an irreversible neurodegenerative disease, and its pathogenesis involves various mechanisms such as neuroinflammation and β-amyloid deposition. Erjing Pills can inhibit neuroinflammation by inhibiting toll-like receptor 4/nuclear factor kappa-B/nucleotide-binding domain leucine-rich repeat and pyrin domain-containing protein 3; however, qualitative analysis of the material basis is lacking. Therefore, it is necessary to analyze and explore the material basis of network pharmacology research. This study employed a multifaceted approach, including drug-like screening, molecular docking, and bioinformatic analysis. Preliminary screening identified 59 drug ingredients in Erjing Pills that met the Absorption, Distribution, Metabolism, Excretion and Toxicity screening criteria. Among these, 7 ingredients, including diosgenin, exhibited superior binding properties compared with the positive drugs in molecular docking. Gene ontology annotation and pathway analysis revealed their involvement in crucial biological processes, such as hormone response, insulin resistance, and steroid hormone biosynthesis signaling pathways, which are known for their anti-inflammatory and cognitive enhancement effects. A meta-analysis of relevant literature corroborated the anti-inflammatory activities of diosgenin and 5 other ingredients. These 5 ingredients, with diosgenin as a prominent candidate, exert anti-inflammatory effects by targeting key components of the toll-like receptor 4/nuclear factor kappa-B/nucleotide-binding domain leucine-rich repeat and pyrin domain-containing protein 3 inflammatory pathway, thereby presenting potential efficacy in the treatment of Alzheimer disease.

## 1. Introduction

Alzheimer disease (AD) is a progressive and irreversible neurodegenerative disease that is characterized by the presence of amyloid plaques and neurofibrillary tangles.^[1]^ The prevalence of AD has been reported to increases with age. It is estimated that there are a total of approximately 46 million people with dementia worldwide, and this number will gradually increase at a rate of 1-fold every 20 years, and is expected to reach 74.7 million by 2030 and 131.5 million by 2050, imposing a serious economic burden on families and society.^[2]^ Existing drugs can control and alleviate AD symptoms to a certain extent, but their therapeutic effects are unsatisfactory. Therefore, it is socially and economically important to search for highly effective drugs to delay and control the occurrence and development of AD.

Erjing Pills are composed of 2 Chinese medicines, Huangjing and Lycium barbarum, which nourish the kidney, nourish yin, and benefit wisdom; it has the therapeutic effects of improving learning memory and inhibiting the loss of nidus vesicles in the CA1 area of the hippocampus in rats with A,^[3]^ and the existing evidence suggests that neuroinflammatory responses, such as the activation of neuroglial cells (e.g., microglia and astrocytes), expression of key inflammatory mediators, and neurotoxic free radicals,^[4]^ play an important role in the pathogenesis of AD. Nonsteroidal anti-inflammatory drugs may reduce the risk of developing AD,^[5,6]^ and long-term use of nonsteroidal anti-inflammatory drugs may delay the onset or slow the progression of AD.^[7,8]^

The toll-like receptor 4 (TLR4)/nuclear factor kappa-B (NFκB)/nucleotide-binding domain leucine-rich repeat and pyrin domain-containing protein 3 (NLRP3) inflammatory pathway, as a classical neuroinflammatory pathway, was found in a pre-laboratory study that Erjing Pills could downregulate the expression of NFκB p65, p-NFκB p65, IκBα, and p-IκBα in D-galactose-combined with Aβ_25–35_ composite modeling-induced AD rats, thus exerting an inhibitory neuroinflammation effect through the inhibition of TLR4/NFκB/NLRP3 and the reduction of downstream inflammatory factors^[9]^; however, the material basis for the prevention and treatment of AD and the mechanism of action are yet to be known.

Through the study of existing published works, Yang et al,^[10]^ using the means of network pharmacology, to AD multiple disease targets as the target of the material basis of the study, and ultimately did not clarify the specific signaling pathway of Erjing Pills to prevent and control AD, the depth of research on the mechanism of action is relatively shallow, this paper, in the early stage of the clear anti-inflammatory effect of Erjing Pills, on the basis of the study, to explore the role of Erjing Pills TLR4/NFκB/NLRP3 inflammatory pathway, combined with the verification of the later components, to further clarify the material basis and mechanism of action of Erjing Pills in preventing and treating AD, and to provide scientific basis for the clinical prevention and treatment of AD by Erjing Pills.

The purpose of this study is to integrate various research tools such as drug-like properties screening, molecular docking, intermolecular force analysis, gene ontology annotation (GO annotation) analysis, Kyoto Encyclopedia of Genes and Genomes (KEGG pathway analysis), etc, and on the basis of the research that Erjing Pills has an anti-inflammatory effect in the early stage, to explore the main active components and targets of Erjing Pills in the TLR4/NFκB/NLRP3 inflammatory pathway, and combined with the verification of the components of Erjing Pills in the later stage, to clarify the material basis and mechanism of action for the anti-AD effect of Erjing Pills, and to provide new alternatives for further research and development of drugs with anti-AD efficacy. Combined with the validation of the components of Erjing Pills at a later stage, the material basis and mechanism of action of Erjing Pills will be clarified to provide new alternatives for further research and development of drugs with efficacy in preventing and treating AD, so that patients with AD can obtain health at an early date, and also to provide a new and feasible research tool for modern research on Traditional Chinese Medicine (TCM), which can integrate more technological means of research and further increase the depth of research on the material basis and mechanism of Chinese medicine.

## 2. Methods

### 2.1. Software

The software used in this study included Discovery Studio Client 4.5, Autoduck Vina 1.5.6^[11]^ and OpenBabel 2.4.1.

### 2.2. Acquisition and screening of chemical composition of Erjing Pills

The active ingredients in Erjing Pills were screened from the literature (search terms: “Erjing Pills,” “Polygonatum sibiricum” and “Lycium chinense”),^[12,13]^ TCM Database@Taiwan (http://tcm.cmu.edu.tw/), TCM Systems Pharmacology Database (http://tcmsp-e.com/tcmsp.php), Chinese Academy of Sciences chemical database (http://www.organchem.csdb.cn), and the collected ingredients were screened and analyzed for drug likeness using Discovery Studio software. Pharmacokinetic properties, including absorption, distribution, metabolism, excretion, and toxicity, are major factors that influence biological activity. In the present study, 5 Absorption, Distribution, Metabolism, Excretion and Toxicity (ADMET)-related parameters, aqueous solubility, blood, brain, barrier penetration, human intestinal absorption, plasma protein binding, and hepatotoxicity, were used to identify the potential bioactive components of Erjing Pills. The screening criteria for active components in Erjing Pills were as follows: intestinal absorption: 0 to 1; water solubility, −6 to 0; blood–cerebrospinal fluid barrier permeability: 2 to 4; cytochrome P4502D6 inhibition, FALSE; and hepatotoxicity, FALSE.

### 2.3. Docking preprocessing of the chemical components of the Erjing Pills

The Erjing Pill compositions obtained in Section 2.2, which satisfy the ADMET screening conditions, were processed using AutoDockTools for hydrogenation, addition of Gasteriger charge and atom type, and Choose Torsions to select the rotatable key and saved as a file in PDB format. The processed Erjing Pills compositions were transferred to pdbqt format using OpenBabel to facilitate the next step of molecular docking. sperm pellet composition in pdbqt format using the OpenBabel software.

### 2.4. Target collection and preprocessing

Target proteins closely related to the TLR4/NFκB/NLRP3 inflammatory pathway were selected from the PDB database (https://www.rcsb.org/), and the ligand expansion method was applied to identify the active sites based on the literature description of the target proteins and co-crystallized small-molecule ligands in the PDB database.^[14]^

### 2.5. Molecular docking

Classical nonsteroidal anti-inflammatory drugs were selected as positive references, such as salicylic acid (aspirin), aniline (acetaminophen), indole (indomethacin), arylacetic acid (diclofenac), and arylpropionic acid (ibuprofen), which were dehydrogenated and hydrogenated in PDB format and then saved in pdbqt format using AutoDock Tools. The center coordinates of the docking box and box size of the molecular docking were adjusted according to the redock situation of the original ligand. If there was no original ligand, then the whole protein was used as the docking space to perform blind docking (blind docking), and the default values of the docking parameters were recorded in the config file: exhaustiveness = 8, energy_range = 3, num_modes = 9. The docking center coordinates and box size parameters are listed in Table 1.

**Table 1:** Molecular docking parameters.

Target protein	Uniprot ID	PDB ID	X	Y	Z	Vina_Box
Toll-like receptor 4	O00206	3FXI	17.52	-4.52	15.1	40 × 40 × 40
MyD88	Q99836	4EO7	12.86	0.58	10.85	40 × 40 × 40
IKKα	O15111	5TQY	159.434	104.434	90.168	50 × 60 × 65
IKKβ	O14920	4KIK	52.595	32.899	-58.611	45 × 40 × 40
IKBα	P25963	1IKN	31.226	24.771	12.112	60 × 60 × 60
Transcription factor p65	Q04206	1NFI	-3.957	52.593	-5.122	40 × 50 × 105
NLRP3	Q96P20	6NPY	85.374	90.417	88.074	50 × 50 × 55
Caspase-1	P29466	6F6R	14.47	33.556	-1.831	60 × 60 × 60
Interleukin-1 β	P01584	1ITB	41.03	8.25	12.35	85 × 55 × 45
iNOS	P35228	4NOS	-1.539	95.559	17.402	50 × 60 × 50
COX2	P35354	5KIR	30.27	11.268	35.116	70 × 60 × 60

COX2 = cytochrome oxidase subunit 2, IKBα = NF-kappa-B inhibitor α, IKKα = inhibitor of kappa B kinase α, IKKβ = inhibitor of kappa B kinase β, iNOS = inducible nitric oxide synthase, MyD88 = myeloid differentiation primary response gene 88, NLRP3 = nucleotide-binding domain, leucine-rich repeat and pyrin domain-containing protein 3.

The docking parameter file is as follows:

receptor = xxx.pdbqt (receptor)

centre_x =

centre_y = (docking center coordinates)

centre_z =

size_x =

size_y = (size of docking box)

size_z =

exhaustiveness = 8

energy_range = 3 (default parameter values for docking)

num_modes = 9

### 2.6. Analysis of docking results

AutoDock Vina’s analysis of ligand and receptor binding was determined by the change in the binding free energy ∆. The scoring function stipulates that when the binding energy affinity is <0, the conformation of the ligand and receptor molecules can be stably bound, and the larger the absolute value of the affinity, the greater the stability. Components with docking binding energies less than all positive drugs were screened as the proposed active ingredients for the action of the TLR4/NFκB/NLRP3 inflammation pathway of Erjing Pills.^[15]^ The binding modes of the target proteins with the highest average value of docking binding energy were also analyzed using Discovery Studio to clarify the form of intermolecular forces present.

### 2.7. Active ingredient target screening

Swiss Target Prediction was used to screen potential targets of the proposed active ingredient by setting the attribute as “homo sapiens.” components were screened as potential targets of the active ingredients.

### 2.8. Pathway enrichment analysis

The screened potential targets were put into the Metascape platform, and after submission, the input species and analyzed species were selected as “H. sapiens,” and the *P* < .01 was set, and the GO annotation analysis was performed on the targets. GO annotation and pathway analyses were performed on the target genes.

### 2.9. Validation of docking results

The components of Erjing Pills cited in the previous molecular docking were obtained from the database and published literature, and basic experiments have proven that Erjing Pills can alleviate the pathological changes of AD by reversing microglial activation and reducing the expression level of neuroinflammatory factors. Therefore, pharmacodynamic experiments were carried out on the screened active components by means of a literature search and database verification to further clarify the material basis for the prevention and treatment of AD by inhibiting the neuroinflammatory pathway. Pharmacodynamic validation of the screened active ingredients will be performed to further clarify the material basis of Erjing Pills in preventing and treating AD by inhibiting neuroinflammation. The databases searched were PubMed, China Knowledge, and Wanfang, and the keywords used were “inflammation” and the proposed active ingredients in the prescreening.

## 3. Results

### 3.1. Pharmacological screening of the ingredients of Erjing Pills

**Figure 1.**
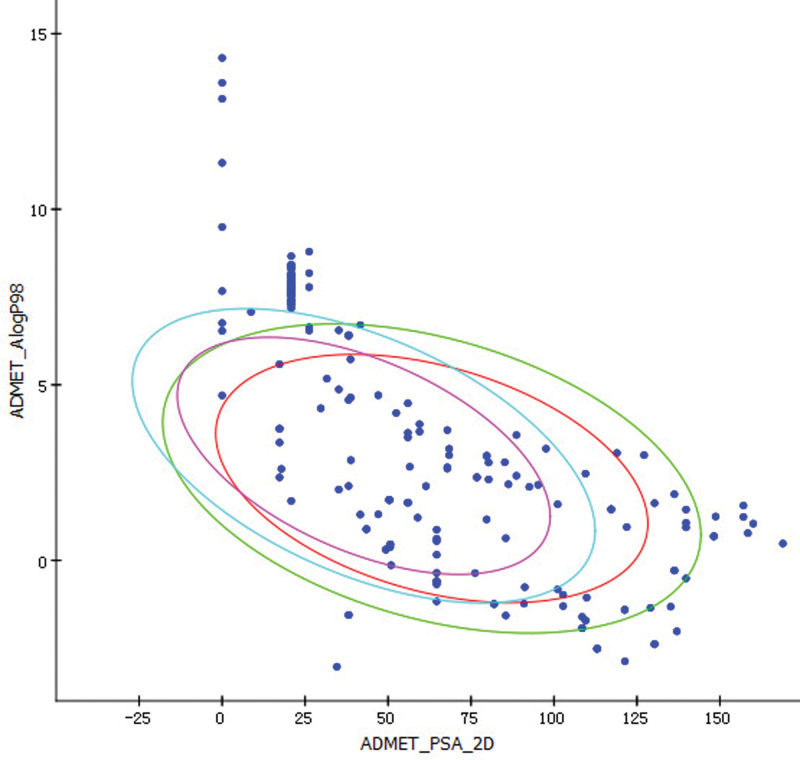
Descriptors: the screening results of ADMET. ADMET_Alog P98 represents the lipid water partition coefficient and ADMET_PSA_2D represents the polar molecular surface area. The 2D graphs of ADMET_PSA_2D and ADMET_Alog P98 display 2 pairs of ellipses, representing the 95% and 99% confidence zones of the blood–brain barrier penetration model (purple and blue) as well as the 95% and 99% confidence intervals of the human intestinal absorption model (red and green). PSA, polar surface area.

A total of 294 compounds were obtained through ingredient collection and the elimination of duplicates. Using the ADMET Descriptors module in the DISCOVERY STUDIO software, the collected ingredients were screened for drug-like properties and 59 compounds, including lauric acid and nicotinic acid, were obtained within the confidence interval (Fig. 1 and Table 2).

**Table 2:** ADMET compliant compounds in Erjing Pills.

Number	Component name	Source	Number	Component name	Source
EJP004	Lauric acid	TCMSP	EJP166	Apigenin	TCMSP
EJP008	Nicotinic acid	TCMSP	EJP171	(+)-Syringaresinol	TCMSP
EJP011	Physcion	TCMSP	EJP172	Diosgenin	TCMSP
EJP013	DBP	TCMSP	EJP173	HMF	TCMSP
EJP017	Paeonol	TCMSP	EJP174	Isoliquiritigenin	TCMSP
EJP035	Atropine	TCMSP	EJP175	Salicylic acid	TCMSP
EJP039	Solavetivone	TCMSP	EJP178	4’,5-Dihydroxyflavone	TCMSP
EJP044	Atropine	TCMSP	EJP179	2’,7-Dihydroxy-3’,4’- dimethoxyisoflavane	TCMSP
EJP048	Ethyl anisate	TCMSP	EJP180	4-Methylolfurfural	TCMSP
EJP049	3-Hydroxy-β-ionone	Shanghai Database	EJP184	Sibiricoside A_qt	TCMSP
EJP054	6-Methoxy-7- hydroxycoumarin	Shanghai Database	EJP186	Sibiricoside B_qt	TCMSP
EJP072	Atropine	Shanghai Database	EJP189	Zhonghualiaoine 1	TCMSP
EJP073	Baogongteng B	Shanghai Database	EJP195	Neosibiricoside D	Literature
EJP089	Hyoscyamine	Shanghai Database	EJP196	(25S)-Pratioside D1	Literature
EJP090	Isoleucine	Shanghai Database	EJP202	Huangjingenin	Literature
EJP095	Leucine	Shanghai Database	EJP206	Huangjinoside F	Literature
EJP100	Nicotinic acid	Shanghai Database	EJP222	spirost-5-en-12-one-3-O-β-D- glucopyranosyl-(1→2)-[β- Dxylopyranosyl-(1→3)]- β-D-glucopyranosyl-(1→4)- β-Dgalactopyranoside	Literature
EJP102	Phenylalanine	Shanghai Database			
EJP105	Safranal	Shanghai Database	EJP230	(25R)-Spirost-5-en-3β,17α-diol-3- O-β-D-glucopyranosyl -(1→3)-[α-L-rhamnopyranosyl- (1→2)]-β-D-glucopyranoside	Literature
EJP106	Scopoletin	Shanghai Database			
EJP108	Solavetivone	Shanghai Database	EJP234	Dioscin	Literature
EJP111	Tryptophane	Shanghai Database	EJP272	New glycyrrhizin	Literature
EJP112	Tyrosine	Shanghai Database	EJP273	Glycyrrhetinic acid	Literature
EJP113	Valine	Shanghai Database	EJP275	New isoglycyrrhizin	Literature
EJP116	β-Ionone	Shanghai Database	EJP276	4’,7-Dihydroxy-3’-methoxyisoflavone	Literature
EJP118	(-)-4’-O-Methyl-nyasol	Taiwan Database	EJP278	2’,7-Dihydroxy-3’,4’- dimethoxyisoflavane glucoside	Literature
EJP129	Atropine	Taiwan Database	EJP279	(6aR,11aR)-10-Hydroxy-3,9- dimethoxypterocarpane>	Literature
EJP134	Cinnamic alcohol	Taiwan Database	EJP280	Polygonatine A	Literature
EJP142	Glycitein	Taiwan Database	EJP281	Polygonatine B	Literature
EJP149	Safrole	Taiwan Database	EJP282	Kinganone	Literature
EJP157	Zederone	Taiwan Database			

ADMET = Absorption, Distribution, Metabolism, Excretion and Toxicity, EJP = Erjing Pills, TCMSP = Traditional Chinese Medicine Systems Pharmacology Database and Analysis Platform.

### 3.2. Molecular docking results

From a docking binding energy perspective, among the inflammatory proteins of the TLR4/NFκB/NLRP3 pathway, the positive reference drug diclofenac had the lowest docking binding energy, followed by acetaminophen, indomethacin, Erjing Pills, and ibuprofen.

**Figure 2.**
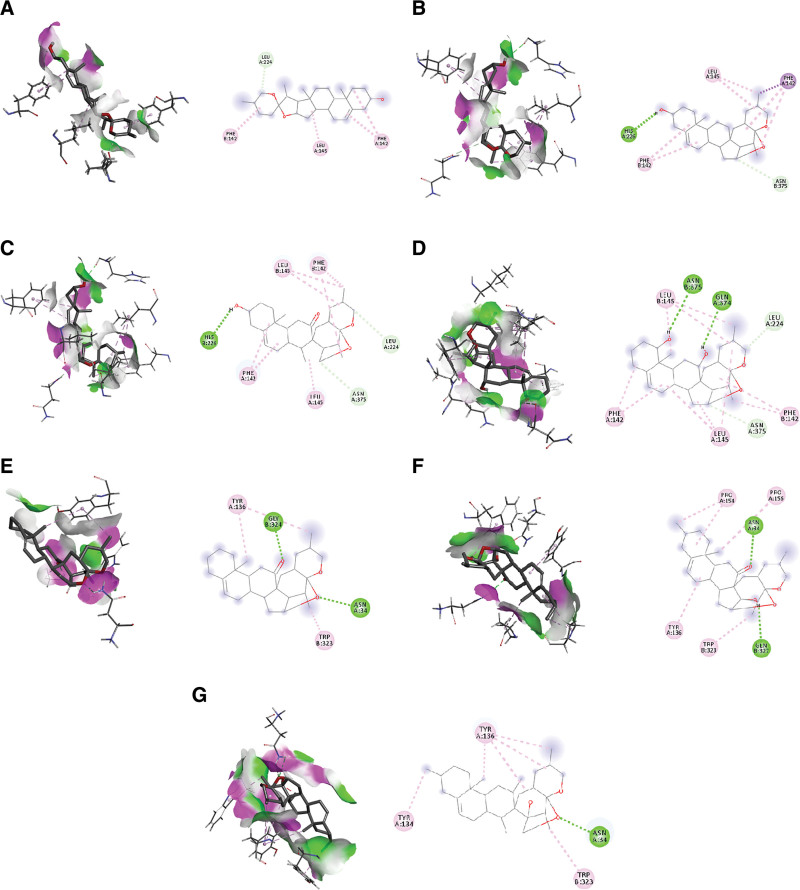
3D and 2D interaction patterns of 7 candidate components with COX2 targets. (A) EJW172, (B) EJW195, (C) EJW196, (D) EJW202, (E) EJW222, (F) EJW230, and (G) EJW234. The receptor residue under the pink surface is an H-bond donor (donor) and the receptor residue under the cyan surface is an H-bond acceptor (acceptor). Intermolecular interactions: light green dashed line, carbon hydrogen bonding; dark green dashed line, conversion hydrogen bonding; pink dashed line, Pi-alkyl; purple dashed line, Pi-Sigma.

Of the 59 constituents of Erjing Pills eligible for ADMET screening, only diosgenin, neosibiricoside D, (25S)-pratioside D1, huangjingenin, spirost-5-en-12-one-3-O-β-D-glucopyranosyl-(1→2)-[β-Dxylopyranosyl-(1→3)]-β-D-glucopyranosyl-(1→4)-β-Dgalactopyranoside,(25R)-spirost-5-en-3β,17α-diol-3-O-β-D-glucopyranosyl-(1→4), (25R)-spirost-5-en-3β,17α-diol-3-O-β-D-glucopyranosyl-(1→3), (25R)-spirost-5-en-12-one-3-O-β-D-glucopyranosyl-(1→3), [α-α-β-D-glucopyranosyl-(1→3)],[α-α-α-glucopyranoside D1 →3)-[α-L-rhamnopyranosyl-(1→2)]-β-D-glucopyranoside, and dioscin, the docking binding energies of the 7 constituents were less than those of all the positive drugs (Table 3). To further assess the binding ability of the 7 candidate compounds to the target proteins of the TLR4/NFκB/NLRP3 inflammatory pathway, the COX2 group, which had the highest average docking binding energy, was selected to analyze the binding patterns and clarify the existence of intermolecular forces (Fig. 2).

**Table 3:** Molecular docking results (binding energy kcal/mol).

Composition	PDB ID and combining energy										
	1IKN	1ITB	1NFI	3FXI	4EO7	4KIK	4NOS	5KIR	5TQY	6F6R	6NPY
Aspirin	−5.9	−5.5	−6.3	−6.9	−5.7	−5.9	−6.2	−6.9	−5.6	−6.2	−6.3
Paracetamol	−7.0	−6.6	−6.2	−6.7	−5.6	−6.4	−7.3	−7.4	−6.8	−5.9	−7.4
Indometacin	−6.6	−6.7	−6	−5.8	−6.7	−6.9	−7.7	−6.8	−6.9	−6.2	−6.8
Diclofenac	−8.2	−7.3	−7.5	−7.3	−7.8	−7.1	−8.4	−8.6	−7.7	−7.8	−9.0
Ibuprofen	−6.1	−6.2	−5.9	−5.3	−5.2	−5.7	−6.2	−5.7	−5.2	−5.2	−5.5
ABE of the positive drug	−6.8	−6.5	−6.4	−6.4	−6.2	−6.4	−7.2	−7.1	−6.4	−6.3	−7.0
EJP172	−9.4	−9.3	−9.4	−9.2	−8.3	−7.8	−10.0	−11.0	−9.6	−8.7	−9.6
EJP195	−9.0	−9.0	−9.1	−9.5	−9.1	−8.5	−9.6	−12.1	−9.0	−8.7	−10.7
EJP196	−8.9	−8.7	−8.7	−9.5	−8.5	−8.5	−10.0	−11.0	−9.4	−9.0	−9.0
EJP202	−9.2	−8.8	−9.1	−9.6	−8.4	−8.6	−10.2	−10.6	−9.2	−9.0	−10.6
EJP222	−9.1	−8.9	−9.9	−9.7	−8.9	−8.9	−10.2	−9.7	−9.2	−9.4	−9.8
EJP230	−8.9	−9.0	−9.4	−9.7	−8.3	−9.1	−10.0	−9.9	−10.5	−9.5	−9.7
EJP234	−8.8	−8.9	−9.4	−9.7	−8.7	−8.9	−10.1	−10.1	−9.9	−9.1	−9.5
ABE of candidate compounds	−9.0	−9.3	−7.4	−9.6	−8.6	−8.6	−10.0	−10.6	−9.5	−9.1	−9.8

ABE = average binding energy, EJP = Erjing Pills, PDB = Protein Data Bank.

Small molecules are surrounded by binding-site amino acid residues represented by amino acid character abbreviations and amino acid sequence numbers in the protein. Different colors of dotted lines represent different binding modes of the receptor–ligand; for example, green dotted lines represent hydrogen bonding interactions, mauves are hydrophobic interactions, yellow lines represent charge interactions, etc.

### 3.3. Target screening and pathway enrichment analysis of active ingredients

**Figure 3.**
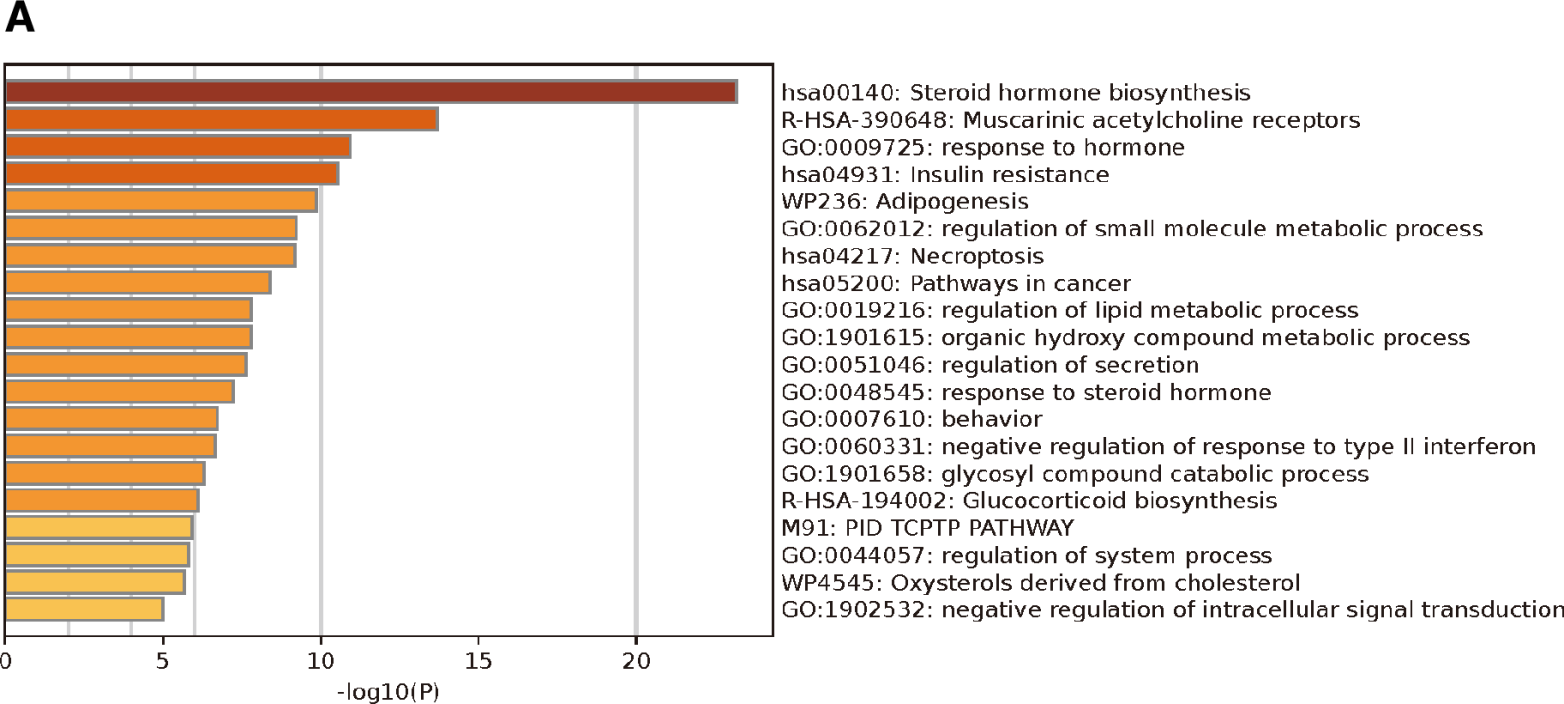
Bar graph of enriched terms across input gene lists. The bar graph represents the number of genes involved in biological processes.

**Figure 4.**
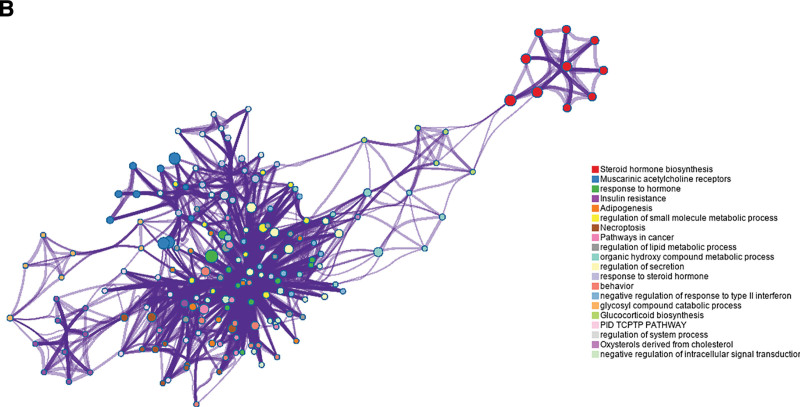
Network of enriched terms. The dots represent the names of the pathways and the line segments represent the connections between the pathways and the pathways.

**Figure 5.**
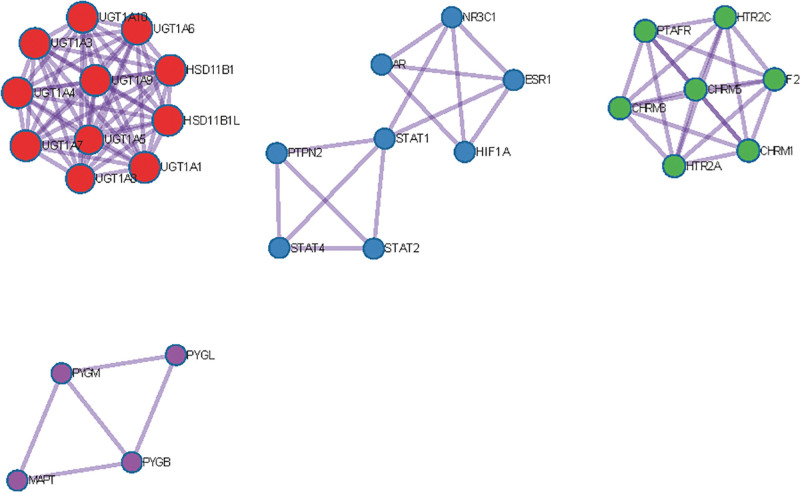
Protein–protein interaction network. Red circle = MCODE1, blue circle = MCODE 2, green circle = MCODE 3, purple circle = MCODE 4.

**Figure 6.**
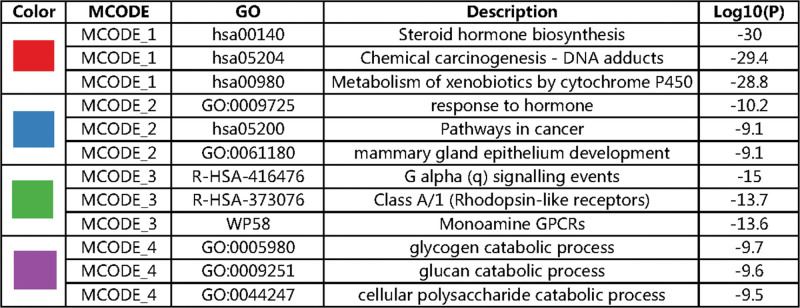
MCODE components identified in the gene lists. Red circle = MCODE1, blue circle = MCODE 2, green circle = MCODE 3, purple circle = MCODE 4.

Using the Swiss Target Prediction platform, 105 potential targets of the active ingredients were screened, and the following pathway and process enrichment analyses were performed using the Metascape platform: KEGG Pathway, GO Biological Processes, Reactome Gene Sets, Canonical Pathways, CORUM, WikiPathways, and PANTHER Pathway (Table 4 and Figs. 3–6).

**Table 4:** Top 20 clusters with their representative enriched terms (one per cluster).

GO	Category	Description	Count	%	Log10 (*P*)	Log10 (q)
hsa00140	KEGG Pathway	Steroid hormone biosynthesis	13	22.81	−23.16	−18.81
R-HSA-390648	Reactome Gene Sets	Muscarinic acetylcholine receptors	5	8.77	−13.7	−10.65
GO:0009725	GO Biological Processes	Response to hormone	15	26.32	−10.94	−8.1
hsa04931	KEGG Pathway	Insulin resistance	8	14.04	−10.53	−7.78
WP236	WikiPathways	Adipogenesis	8	14.04	−9.85	−7.16
GO:0062012	GO Biological Processes	Regulation of small molecule metabolic process	10	17.54	−9.22	−6.59
hsa04217	KEGG Pathway	Necroptosis	8	14.04	−9.18	−6.57
hsa05200	KEGG Pathway	Pathways in cancer	11	19.3	−8.4	−5.85
GO:0019216	GO Biological Processes	Regulation of lipid metabolic process	9	15.79	−7.79	−5.31
GO:1901615	GO Biological Processes	Organic hydroxy compound metabolic process	10	17.54	−7.78	−5.31
GO:0051046	GO Biological Processes	Regulation of secretion	11	19.3	−7.63	−5.16
GO:0048545	GO Biological Processes	Response to steroid hormone	8	14.04	−7.21	−4.79
GO:0007610	GO Biological Processes	Behavior	10	17.54	−6.73	−4.37
GO:0060331	GO Biological Processes	Negative regulation of response to type II interferon	3	5.26	−6.65	−4.31
GO:1901658	GO Biological Processes	Glycosyl compound catabolic process	4	7.02	−6.29	−4.01
R-HSA-194002	Reactome Gene Sets	Glucocorticoid biosynthesis	3	5.26	−6.12	−3.87
M91	Canonical Pathways	PID TCPTP PATHWAY	4	7.02	−5.92	−3.7
GO:0044057	GO Biological Processes	Regulation of system process	9	15.79	−5.81	−3.62
WP4545	WikiPathways	Oxysterols derived from cholesterol	4	7.02	−5.68	−3.52
GO:1902532	GO Biological Processes	Negative regulation of intracellular signal transduction	8	14.04	−5	−2.92

“Count” is the number of genes in the user-provided lists with membership in the given ontology term. “%” is the percentage of all of the user-provided genes that are found in the given ontology term (only input genes with at least 1 ontology term annotation are included in the calculation). “Log10(P)” is the *P*-value in log base 10. “Log10(q)” is the multi-test adjusted *P*-value in log base 10.

### 3.4. Results of docking validation

With the keywords of “inflammation/inflammation,” diosgenin and other 7 proposed active ingredients, the database literature search was carried out in Pubmed, Web of Science, ScienceDirect, China Knowledge Network, and a total of 8900 articles of related literature were retrieved, among which, dioscin was reported to have certain anti-inflammatory activity and inhibitory effect on inflammation-related factors. Diosgenin has been clearly reported in the literature to have certain anti-inflammatory activity, and has a certain inhibitory effect on inflammation-related factors.

Yao et al found that dioscin reduced the overexpression of TLR4 and myeloid differentiation primary response gene 88 in LPS-induced inflammatory mice and rats, resulting in a significant reduction in the levels of downstream inflammatory factors, such as TRAF6, p-NFκB, IL-1β, IL-6, and TNF-α, which in turn reduced inflammatory injury.^[16]^ Tsukayama et al demonstrated that diosgenin inhibits COX2 expression in human non-small cell lung cancer A549 cells and downregulates COX2 and prostate E2 synthase-1 (mPGES-1) expression in a mouse model of lipopolysaccharide (LPS)-induced liver injury. Diosgenin significantly inhibited lipid accumulation and pro-inflammatory cytokine levels in THP-1 macrophages, and significantly downregulated Src and STAT3 phosphorylation.^[17,18]^

## 4. Discussion

TCM is an important component of China’s medicine and healthcare industry, and plays a key role in national disease prevention and treatment. Multiple active ingredients in TCM act on molecular target groups to regulate key biological processes involved in disease development and restore the organism to a state of equilibrium, thus playing a therapeutic role. Currently, drug development based on a multi-target, multi-pathway regulation mode that plays a therapeutic role in disease has become a hot spot and frontier in the field of drug research. Network pharmacology, as the frontier of the discipline of TCM research, integrates the ideas of systems biology and multidirectional pharmacology and is applied to many fields of life science, such as the discovery of lead compounds, the study of the mechanism of action, and the screening of the material basis.^[19]^ Considering the holistic regulation of multiple targets based on the analysis of ADMET drug-forming properties, component–target docking, and the analysis of conformational effects, this approach solves the problems of high cost and long period of time when screening the material basis and also solves the limitations when exploring the mechanism of action. Therefore, network pharmacology is gradually being used to predict the main active ingredients and potential targets of TCM and to elaborate on the material basis and mechanism of action of TCM.^[20,21]^

AD is a progressive and irreversible neurodegenerative disease, and clinical drugs are mostly used to control and alleviate related symptoms; however, their therapeutic effect is not significant. In addition to the oxidative stress hypothesis, the β-amyloid (Aβ) cascade hypothesis, and the tau protein phosphorylation hypothesis, there is increasing evidence suggesting that the neuroinflammation hypothesis is also an important factor in the pathogenesis pathway of AD, in which the TLR4/NFκB/NLRP3 inflammatory pathway, as a classical neuroinflammatory pathway, has received increasing attention in the study of AD pathogenesis.

Erjing Pills are composed of 2 Chinese medicines, Huangjing and Lycium barbarum, which nourish the kidney, nourish yin, and benefit intellect. A previous study found that Erjing Pills have the ability to improve learning and memory in AD rats, which can significantly improve the learning and memory ability caused by de-virginization combined with D-galactose and Aβ_1–40_ injections and reduce the loss of nidus in the hippocampal CA1 area. In this study, we investigated the main active ingredients and target groups of Erjing Pills in the neuroinflammation-associated TLR4/NFκB/NLRP3 pathway using network pharmacology.

In this study, 59 Erjing pill ingredients, including lauric acid and nicotinic acid, which met the ADMET screening criteria, were screened against 12 pathway proteins, including TLR4 in the TLR4/NFκB/NLRP3 inflammatory pathway, to obtain docking binding energies that were lower than the docking binding energies of all positive drugs. Seven ingredients diosgenin, neosibiricoside D, (25S)-pratioside D1, huangjingenin, spirost-5-en-12-one-3-O-β-D-glucopyranosyl-(1→2)-[β-Dxylopyranosyl-(1→3)]-β-D-glucopyranosyl-→4)-β-Dgalactopyranoside,(25R)-spirost-5-en-3β,17α-diol-3-O-β-D-glucopyranosyl-(1→3)-[α-L-rhamnopyranosyl-(1→2)]-β-D-glucopyranoside, and dioscin, suggesting that these 7 ingredients possess stronger affinity for the TLR4/NFκB/NLRP3 pathway and are more prone to develop ingredient–target interactions compared to the 5 positive drugs.

The intermolecular forces mainly consist of hydrogen bonding interactions (conventional hydrogen bonding, carbon–hydrogen bonding, Pi-donor hydrogen bonding), electrostatic interactions (salt bridges, attractive charges, Pi-cations, and Pi-anions), and hydrophobic interactions (Pi-alkyl, alkyl, Pi-Sigma, and Pi-Pi stacking). Analysis of the 2D planar maps of ligand–protein interactions revealed that COX2 and the 7 candidate compounds were mainly bound to the target proteins through hydrogen bonding, carbon–hydrogen bonding, Pi-alkyl, alkyl, and Pi-Sigma binding to the active interaction pockets of the target proteins, forming stable binding of the compounds to the target proteins through hydrogen bonding and hydrophobic interactions, in which hydrogen bonding is the ligand–receptor recognition of each other. Hydrophobic interactions can increase the overall binding affinity of the ligand, which further stabilizes the binding of candidate compounds to target proteins, thus improving the binding ability to amino acid residues at the binding site and affecting the role of the candidate components in the TLR4/NFκB/NLRP3 inflammatory pathway, thereby inhibiting inflammation and improving AD.

In terms of pharmacodynamics, previous studies have shown that Erjing Pills have the effect of inhibiting the expression of transmembrane receptor TLR4, and can downregulate the expression of NFκB p65, p-NFκB p65, IκBα, p-IκBα, in D-galactose combined with Aβ_25–35_ composite modeling-induced AD rats, and thus inhibit the content of downstream inflammatory factors and play a role in inhibiting neuroinflammation by suppressing TLR4/NFκB/NLRP3, and reducing the content of downstream inflammatory factors. Inhibits neuroinflammatory effects. In addition, Erjing Pills significantly reduced IL-1β, TNF-α, and IL-6 inflammation-related indices in hippocampal tissues and inhibited the expression of Aβ_1–42_ and p-Tau^404^ in the hippocampal area, suggesting that Erjing Pills can reverse microglial activation, reduce the expression level of neuroinflammatory factors, and reduce pathological changes in AD.

GO annotation and KEGG pathway analysis of 7 constituents, including diosgenin, revealed that in addition to being involved in biological processes, such as response to hormones, regulation of secretion, organic hydroxy compound metabolic processes, and behavior, they are also involved in multiple mechanistic pathways, such as steroid hormone biosynthesis, insulin resistance, necroptosis, and pathways in cancer processes, it is also related to multiple mechanistic pathways such as steroid hormone biosynthesis, insulin resistance, necroptosis, and cancer pathways. Lu et al demonstrated that responses to biological hormone processes, insulin resistance, and steroid hormone biosynthesis signaling pathways are all related to inflammation and learning memory, suggesting that diosgenin and 7 other components can produce anti-inflammatory effects by participating in relevant biological processes and signaling pathways to produce anti-inflammatory effects and improve learning and memory.^[22,23]^

Finally, the pharmacodynamics of the screened active ingredients were verified by literature search, among which diosgenin and dioscin have clear anti-inflammatory activities and have certain inhibitory effects on inflammation-related factors, neosibiricoside D, (25S)-pratioside D1, and huangjingenin belong to the saponin class compounds. Sung et al found that saponin compounds in huangjingenin have therapeutic effects on memory and inflammation.^[24,25]^ A tentative search through literature databases such as Pubmed for spirost-5-en-12-one-3-O-β-D-glucopyranosyl-(1→2)-[β-Dxylopyranosyl-(1→3)]-β-D-glucopyranosyl-(1→4)-β-D-galactopyranoside, (25R)-spirost-5-en-3β,17α-diol-3-O-β-D-glucopyranosyl-(1→3)-[α-L-rhamnopyranosyl-(1→2)]-β-D-glucopyranoside, anti-inflammatory Related Literature showed that the 5 components of diosgenin, neosibiricoside D, (25S)-pratioside D1, huangjingenin, and dioscin have anti-inflammatory pharmacological activities.

## 5. Conclusion

In summary, the 5 ingredients of diosgenin, neosibiricoside D, (25S)-pratioside D1, huangjingenin, and dioscin in Erjing Pills have anti-inflammatory activities by acting on TLR4, myeloid differentiation primary response gene 88, inhibitor of kappa B kinase α, inhibitor of kappa B kinase β, NF-kappa-B inhibitor α, and other related targets in the TLR4/NFκB/NLRP3 inflammatory pathway to produce anti-inflammatory effects and thus exert therapeutic efficacy in the treatment of AD. Nonetheless, the limitations of network pharmacology necessitate further experimental validation of the preliminary results.

## 6. Future prospects

The article integrates pharmacophore screening, molecular docking, intermolecular force analysis, GO annotation analysis, KEGG pathway analysis, and result validation, and the final results show that the Chinese medicine Erjing Pills can produce anti-inflammatory effects by acting on the relevant targets in the inflammatory pathway, and through the later validation, it is proved that the relevant constituents have the therapeutic efficacy of preventing and controlling AD, which provides new alternatives for the further research and development of medicines with the therapeutic efficacy of preventing and controlling AD. It provides new alternatives for the further development of drugs with AD prevention and treatment effects as well as new research tools for improving the depth of research on the material basis and mechanism of TCM.

## Acknowledgments

This study was supported by the Qilu Institute of Technology Research Program Project (grant number: QIT22NN009).

## Author contributions

**Conceptualization:** Chen Zhang, Mingjing Lu, CunNeng Li, Chao Qi, Qian Lin, Liping Huang.

**Data curation:** Chen Zhang, CunNeng Li, Hailing Ding.

**Formal analysis:** Chen Zhang, Chao Qi, Qian Lin.

**Funding acquisition:** Chen Zhang, Liping Huang.

**Investigation:** Chen Zhang, Mingjing Lu.

**Methodology:** Chen Zhang, Mingjing Lu, CunNeng Li, Chao Qi, Liping Huang.

**Resources:** Chen Zhang.

**Supervision:** Mingjing Lu, Liping Huang.

**Validation:** Chen Zhang, CunNeng Li, Liping Huang, Hailing Ding.

**Visualization:** Chao Qi, Qian Lin, Hailing Ding.

**Writing – original draft:** Chen Zhang.

**Writing – review & editing:** Liping Huang, Hailing Ding.
